# Supplementary material for: Predicting the Exception—CRP and Primary Hip Arthroplasty
Source: J Clin Med. 2021 Oct 27;10(21):4985. doi: 10.3390/jcm10214985 (PMC8584609; doi:10.3390/jcm10214985)
Supplement: Supplementary file 1 [file jcm-10-04985-s001.zip › jcm-1401171-supplementary.pdf]

Supplementary Materials

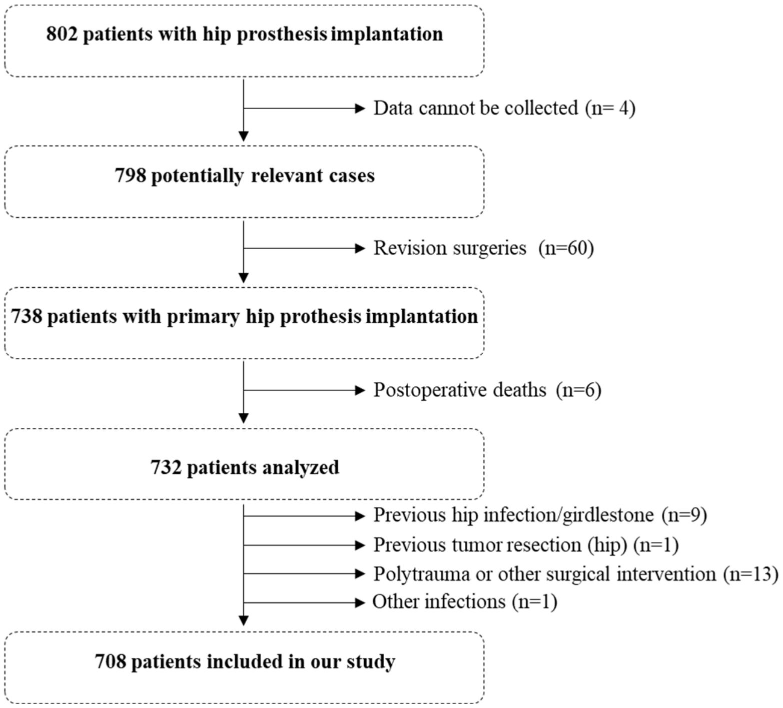

Figure S1. Flowchart for inclusion of patients. From the 802 patients assessed, four contained incomplete data. After removal of primary revision surgery and postoperative deaths, 732 patients were characterized in-depth. From these, 24 patients had to be excluded due to the illustrated criteria.

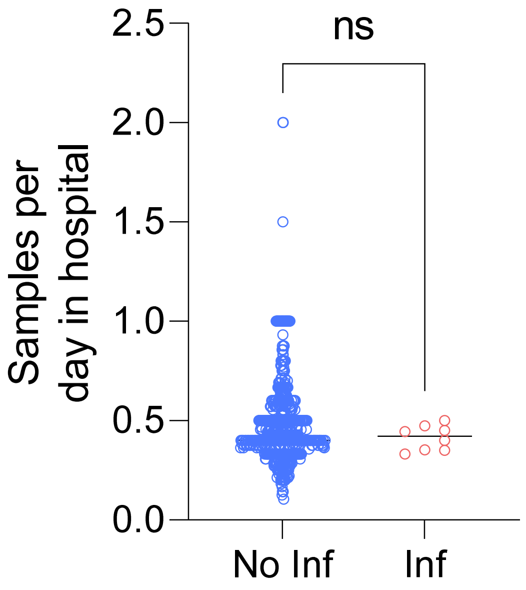

Figure S2. Samples per day in hospital. An average of 0.471 (±0.20) samples were collected per day in hospital in the non-infection group vs. 0.413 (±0.06) in the infection group. There was no difference between the two cohorts (Mann–Whitney, p=0.61).

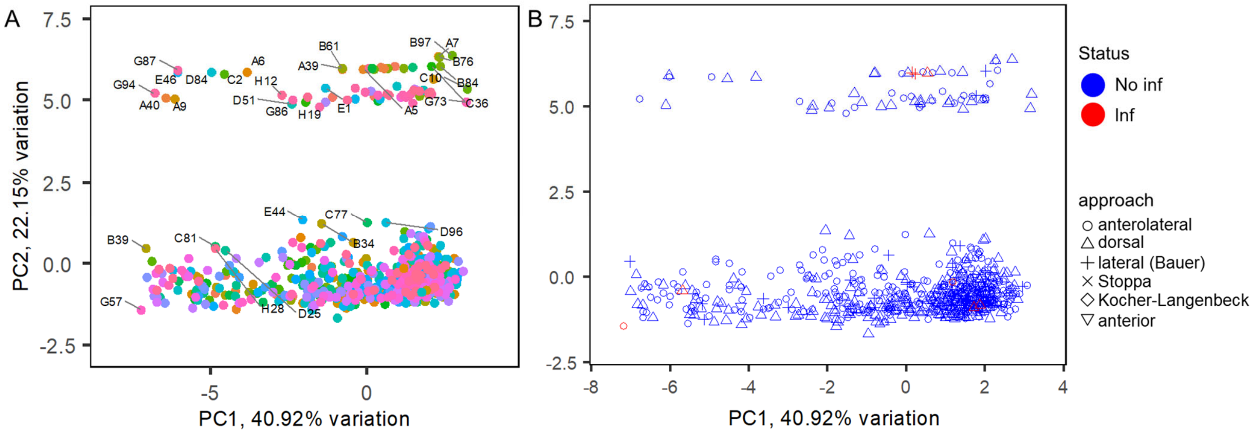

Figure S3. PCA plots summarize all patients, weighed by all assessed parameters to distinguish infectious and non-infectious cohorts. (A) All patients (n=708), weighed by 37 differentiating parameters creates a heterogeneity of two major populations. Out of these, eight patients suffered an infectious complication. (B) The infection status is coded by color, while the approach is coded by shape. Altogether, a combination of all these parameters does not lead to a valid differentiation.
